# Increasing complexity of opsin expression across stomatopod development

**DOI:** 10.1002/ece3.10121

**Published:** 2023-05-26

**Authors:** Sitara Palecanda, Mireille Steck, Megan L. Porter

**Affiliations:** ^1^ School of Life Sciences University of Hawaiʻi at Mānoa Honolulu Hawaii USA

**Keywords:** crustacean, larval development, opsin, transcriptomics, vision

## Abstract

Stomatopods are well studied for their unique visual systems, which can consist of up to 16 different photoreceptor types and 33 opsin proteins expressed in the adults of some species. The light‐sensing abilities of larval stomatopods are comparatively less well understood with limited information about the opsin repertoire of these early‐life stages. Early work has suggested that larval stomatopods may not possess the extensive light detection abilities found in their adult counterparts. However, recent studies have shown that these larvae may have more complex photosensory systems than previously thought. To examine this idea at the molecular level, we characterized the expression of putative light‐absorbing opsins across developmental stages, from embryo to adult, in the stomatopod species *Pullosquilla thomassini* using transcriptomic methods with a special focus on ecological and physiological transition periods. Opsin expression during the transition from the larval to the adult stage was further characterized in the species *Gonodactylaceus falcatus*. Opsin transcripts from short, middle, and long wavelength‐sensitive clades were found in both species, and analysis of spectral tuning sites suggested differences in absorbance within these clades. This is the first study to document the changes in opsin repertoire across development in stomatopods, providing novel evidence for light detection across the visual spectrum in larvae.

## Graphical abstract

Larval stomatopods were thought to have less complex photosensory systems than their adult counterparts; however, their light detection abilities may be quite extensive. Here, we characterized the expression of light‐absorbing opsins across developmental stages of two stomatopod species using transcriptomic methods. The number of opsins expressed increased across ontogeny with all stages expressing opsins from short, middle, and long wavelength‐sensitive clades, providing novel evidence for light detection across the visual spectrum.

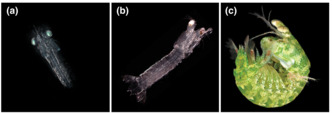

## 1 INTRODUCTION

Stomatopods are known for their quick strikes, both as a means of prey capture and as a method of defense against predators and conspecifics. These abilities are predicated on their highly complex visual systems with up to 12 different retinal photoreceptors dedicated to color detection as well as the ability to detect circularly and linearly polarized light (Chiou et al., 2008; Marshall, 1988; Marshall et al., 2007). Many studies have characterized the unusual diversity of opsins and spectral sensitivities present in this order of pancrustaceans (Bok et al., 2014; Cronin & Marshall, 1989; Porter et al., 2009, 2013; Thoen et al., 2017), but the majority have focused on adults, which only represent a portion of the stomatopod life cycle. Like many other arthropods, stomatopods are indirect developers and pass through several embryonic and larval stages, the exact number of which is dependent on species, before becoming adults (Morgan & Goy, 1987; Morgan & Provenzano, 1979; Williams et al., 1985). While stomatopods spend less time in these early life history stages, they are still incredibly important in terms of dispersal and finding appropriate habitats for the longer adult stage.

Previous studies of larval stomatopod photoreception have focused on vision and the juxtaposition of the simple larval retina with the complex adult one. This is largely due to the unusual way their eyes transition from the larval to the adult stage. While most pancrustaceans modify existing larval structures to form the adult visual system (Douglass & Forward, 1989; Jinks et al., 2002; Meyer‐Rochow, 1975; Nilsson, 1983), stomatopods instead build an adult retina, the portion of the eye where light is detected, alongside but separate from the existing larval retina (Cronin & Jinks, 2001; Williams et al., 1985). During the postlarval phase, the stage at which animals become benthic and begin the transition to juveniles, both the larval and adult retina are functional (Feller et al., 2015), with the larval retina slowly degrading and the adult retina taking its place by the time the animal becomes a juvenile. While the larval retina is spatially homogeneous, adult stomatopods possess an eye that is divided into dorsal and ventral hemispheres with a band of two to six enlarged ommatidia rows between them. This area, known as the midband, is primarily responsible for the color and polarization detection capabilities of adult stomatopods (see review, Marshall et al., 2007). The creation of a new retina is potentially energetically costly (Marshall, 2017; Moran et al., 2015) and suggests that the increase in complexity between the larval and adult stomatopod eye is too great to be accommodated by simple modification.

Early studies of larval stomatopod light sensitivity showed a single peak wavelength absorbance by the retinas of 10 species, with some variation among individuals, which was explained by changes throughout ontogeny (Feller & Cronin, 2016). This aligned with the hypothesis that only one visual pigment existed in larval eyes (Cronin et al., 1995; Jutte et al., 1998), a conclusion which fit with the more visually uniform habitat of these life stages. However, the existence of photonic structures which reflect certain wavelengths of light and help tune the larval visual system in one family of stomatopod (Feller et al., 2019), as well as evidence of ultraviolet sensitivity in another (McDonald et al., 2022), indicate that larval stomatopod visual systems may be capable of detecting more than one peak wavelength.

Nonvisual photoreception has not been studied in larval stomatopods, but it has been reported in other larval invertebrates including arthropods (Cronin et al., 2017; Randel & Jékely, 2016). The expression of visual opsins in the central nervous system of adult stomatopods is correlated with a light‐dependent escape response similar to that found in other pancrustaceans (Donohue et al., 2018; Kingston & Cronin, 2016). The use of nonvisual photoreception by embryonic and larval stomatopods can therefore not be discounted and may be ecologically important for these life stages.

Larval and adult stomatopods live in very different light environments. While adults, particularly from superfamilies Lysiosquilloidea and Gonodactyloidea, live in shallow sand or reef environments under relatively broad spectrum light, their larvae are pelagic and would therefore be subject to an environment that is structurally less complex and resembles an open light field (Losey et al., 1999). While adult stomatopods use color detection to recognize aggressive displays and find mates (Chiou et al., 2011; Franklin et al., 2019), larvae lack these behaviors. Not a lot is known about larval stomatopod ecology, but there is evidence that at least one species is a vertical migrator (Senta, 1967) and that the depths at which it is found change over the course of development with later‐stage larvae found at depths of at least 30 m (Ohtomi et al., 2005). This may act as a mechanism to facilitate dispersal in early stages as has been observed in decapod larvae (Cohen et al., 2015; Epifanio & Tilburg, 2008). Little else is known about how stomatopod larvae locate habitat for settlement, but at least one study has indicated that postlarval stomatopods rely on visual cues among others (Lecchini et al., 2010). Larval stomatopod response to light also shifts throughout development with the earliest larval stage, the propelagic, being negatively phototaxic while later larval stages show positive phototaxis (Dingle, 1969). Like their adult counterparts larval stomatopods are predatory (Harrison et al., 2021) and likely rely on visual cues for prey capture, though definitive studies have not yet been published on this topic.

In order to better understand the limits of larval stomatopod light sensitivity, it is necessary to categorize the molecular components that underlie color detection, opsin proteins. Opsins bind to a vitamin A‐derived chromophore, usually retinal, to form a light‐sensitive photopigment, which absorbs photons and begins the cellular signaling cascade resulting in photoreception (Briscoe, 2001; Nagata et al., 2018). Photopigments preferentially absorb a range of wavelengths of light based on the particular amino acid sequence of their opsin and the vitamin A derivative used as the chromophore. Because species usually use the same chromophore type for all opsin‐containing photopigments, variation in absorbance can be primarily attributed to changes in the opsin's amino acid sequence (Porter et al., 2007). Opsin diversity has been characterized in the eyes of several species of adult stomatopod (Cronin et al., 2010; Porter et al., 2009, 2013, 2020; Valdez‐Lopez et al., 2018) and appears to correspond with the structural complexity of the eye with the number of opsins found appearing higher in eyes with a larger number of unique photoreceptors (Porter et al., 2010, 2013, 2020; Steck, 2016), though more opsin data is needed to determine whether this correlation holds true for all species. The most well characterized stomatopod species, *Neogonodactylus oerstedii*, has 33 identified opsins and 16 photoreceptor types in the eye with co‐expression of opsins in multiple visual photoreceptors, as well as extraocular photoreceptors (Donohue et al., 2018; Porter et al., 2020). Given this trend, we would expect larval stomatopods to express far fewer opsin transcripts than their adult counterparts because they have fewer types of characterized photoreceptors (Feller & Cronin, 2016; Jutte et al., 1998).

Opsin expression in developing embryos has been observed in both vertebrates and invertebrates, preceding the differentiation of photoreceptors (Passamaneck & Martindale, 2013; Saha & Grainger, 1993; Takechi & Kawamura, 2005). *Pullosquilla thomassini* embryos in the final stage before hatching show movement within their egg cases when light is shined on them, and have already developed the characteristic eyeshine associated with larvae of this species (S. Palecanda personal observation; Feller & Cronin, 2014). Thus, we would expect reduced but existent expression of opsins in embryonic stages.

Here we investigate the opsin repertoire of two species of stomatopod at different points during development, allowing us to characterize the temporal sequence of opsin expression across stomatopod life histories. We first focused on a burrowing lysiosquilliod, *Pullosquilla thomassini* (Manning, 1978), which is found in intertidal and subtidal environments. Sampling was conducted across ontogenetic stages to capture the two major transitions in habitat and ecology, from embryo to pelagic larva and from pelagic larva to benthic adult. The results of this detailed investigation indicated that the greatest transition in opsin repertoire for stomatopods occurs at the point when the adult retina is being formed. To determine whether these results would hold true across species, a focused investigation of the larval to adult transition was performed in the locally abundant gonodactyloid, *Gonodactylaceus falcatus* (Forskål, 1775) using improved sequencing technology. The adult and larval visual systems of this species have been previously characterized using microspectrophotometry (MSP; Cronin et al., 1995, 1996), but the opsin repertoire has never been investigated in either stage. *Gonodactylaceus falcatus* is a shallow water species that lives in reef or rubble habitats. This study is the first to quantify the opsin repertoire of stomatopods over the course of their life history, greatly increasing our understanding of how changes in the ability to detect different wavelengths of light may help these animals adapt to different conditions over the course of development. Like their adult counterparts, larval stomatopods likely rely on photoreception for a variety of important behaviors including remaining in the parental burrow until they have developed sufficient swimming abilities, hunting, and finding a habitat for settlement (Dingle, 1969; Harrison et al., 2021; Lecchini et al., 2010). Understanding more about larval stomatopod light perception can further inform our understanding of these key behaviors and the signals that drive them.

## 2 METHODS

### 2.1 Collection and identification

Sampling of *P. thomassini* was conducted in July 2013 and September 2015 at the Lizard Island Research Station, off the eastern coast of Queensland Australia. Adult animals and egg clutches were collected in clear water subtidal habitat (0.5–1 m depth) using a yabby pump to draw up whole sand burrows. Sand was sifted through a 2‐mm metal sieve, and adult animals and egg clutches were removed. Egg clutches were kept only if they were found with an adult which was later identified using morphology to ensure eggs were from the target species. Animals were kept in filtered sea water at room temperature (~23°C) under natural light conditions for up to a week prior to being sacrificed and eyes being fixed. Embryos were cared for by adult animals until they reached the appropriate stage for fixation. Propelagic larvae, the stage directly after hatching which remain in the parental burrow, were collected from egg clutches, which hatched in captivity and fixed whole within 24 h of hatching. Pelagic, postbrooding, larval samples were collected close to shore (0–1 m depth) using dip nets. Collections took place after sunset and dive lights (TUSA TUL‐1000) were used to attract larvae, as stomatopods are positively phototaxic in their pelagic stages (Dingle, 1969). Stomatopod larvae were sorted by approximate stage and fixed whole immediately following collection. All animals and tissue were fixed in RNALater (Invitrogen). Samples were kept at 4°C overnight and then held at −20°C until they were transported to the University of Hawaiʻi at Mānoa where they were moved to −80°C until RNA extractions were performed.

*Gonodactylaceus falcatus* larvae and adults were collected on the Hawaiian island of Oʻahu, USA between February 2016 and February 2017. Adults were collected using dip nets in clear shallow water (0.5–1 m depth) at Wailupe beach park (GPS coordinates: 21.276208, −157.760632). Animals were sacrificed and eyes were removed for fixation within 24 h of capture. Larvae and postlarvae were collected close to shore (0– 1 m depth) at the Hawaii Institute of Marine Biology in Kāneʻohe Hawaiʻi, using the same methods as in previous larval sampling.

Adult animals were identified to species using morphology (Ahyong, 2001; Schram & Muller, 2004), and their sex was determined. Embryonic *P. thomassini* samples were identified based on the adults each egg clutch was collected with, as adults of this species are egg brooders. Larval and postlarval samples were given a preliminary identification using morphology (K. Feller personal communication; Jutte, 1997; Shanbhogue, 1978) and identities were confirmed using molecular barcoding of cDNA. A superscript IV reverse transcriptase kit (Invitrogen) was used with RNA extracted using protocols detailed below to produce cDNA which was then amplified following established protocols for COI barcoding of larval mantis shrimp (Palecanda et al., 2020). PCR amplicons were cleaned using EXO‐SAP‐IT (Thermo Fisher) and sequenced at the Advance Studies in Genomics, Proteomics, and Bioinformatics facility at the University of Hawaiʻi at Mānoa (Honolulu, HI, USA). Nucleotide sequences were identified using the National Center for Biotechnology Information's (NCBI) Basic Local Alignment Tool (BLASTn) and searched against the nucleotide collection (nr/nt) database. Species identities were determined by a percent identity of 99% or higher. Both of the species sampled here are well represented in the NCBI database, and no individuals had ambiguous matches.

**FIGURE 1:**
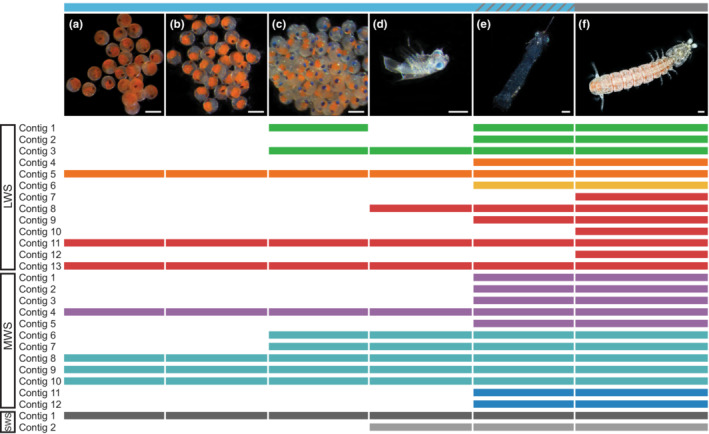
Representative photos of *Pullosquilla thomassini* developmental stages with retina type indicated by bar on top. (a) embryonic stage 1, (b) embryonic stage 2, (c) embryonic stage 3, (d) propelagic larva (K. Feller), (e) late‐stage larva, (f) adult female (R. Caldwell). All scale bars represent 1 mm. Top bar blue = larval retina, and gray = adult retina. Lower bars represent the stages at which each opsin transcript was expressed and divided into long (LWS), middle (MWS), and short (SWS) wavelength‐sensitive clades. All photos © S. Palecanda except where otherwise stated.

**FIGURE 2:**
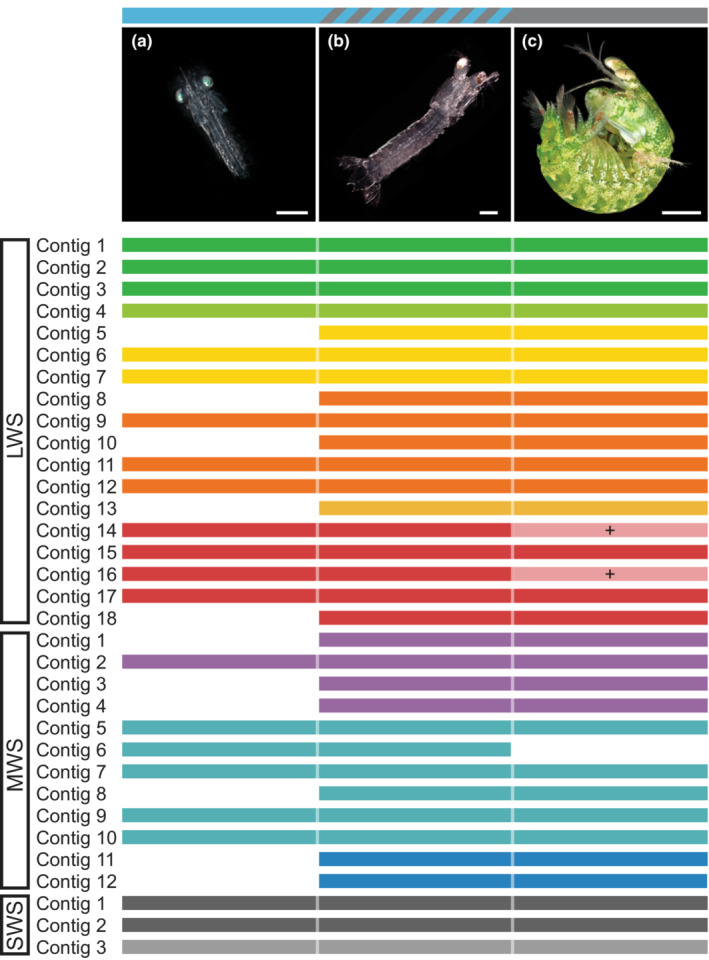
Representative photos of *Gonodactylaceus falcatus* developmental stages with retina type indicated by bar on top. (a) stage four larva (K. Feller), (b) postlarva (K. Feller), (c) adult (R. Caldwell). Scale bars represent 1 mm for (a,b), 1 cm for (c). Top bar blue = larval retina, and gray = adult retina. Lower bars represent the stages at which each opsin transcript was expressed and divided into long (LWS), middle (MWS), and short (SWS) wavelength‐sensitive clades. Opsins that were found in the adult female but not adult male retina are indicated by a + sign.

Approximate stage was determined based on body size and retinal development for *P. thomassini* embryos and larvae (Figure 1). Embryonic samples are referred to in this study as embryonic stages 1, 2, and 3, characterized by the appearance of eye spots, the separation of retinal pigment from the developing body, and the presence of eyeshine caused by the reflection of light from structures in the larval eye (Feller & Cronin, 2014), respectively. The propelagic stage refers to larvae directly after they emerge from eggs, late stage refers to larvae nearing metamorphosis to juveniles. *Gonodactylaceus falcatus* larvae were determined to be stage four or five based on their lack of remaining yolk, vigorous swimming behavior, and morphology as determined by Shanbhogue (1978), while the postlarval stage was identified based on juvenile morphology and the presence of a double retina (Williams et al., 1985; Figure 2).

### 2.2 Transcriptome sequencing and assembly

RNAlater preserved samples were transported to the University of Hawaiʻi for processing. Total RNA was extracted using a RNeasy Mini Kit (Qiagen) following manufacturer protocols. An on‐column DNase digestion was performed to remove residual DNA. RNA was quantified using a Qubit (3.0) fluorometer. Because of low RNA yield and cost of sequencing, one transcriptome from each developmental stage and species was sequenced. Embryonic and larval samples consisted of pooled RNA from ~200 eggs, ~60 propelagic larval whole bodies, or two early‐stage larval whole bodies. Late‐stage larval samples consisted of pooled RNA from two heads. Postlarval samples consisted of a single head and adult samples consisted of two eyes from the same individual.

*Pullosquilla thomassini* samples were sequenced by The California Institute for Quantitative Biosciences at the University of California at Berkeley (Berkeley, CA, USA) using the Illumina HiSeq 4000. *Gonodactylaceus falcatus* samples were sequenced by Novogene using Illumina NovaSeq 6000 technology. Both methods produced paired‐end 150 bp reads and utilized multiplexing of libraries to cut down on cost and provide similar sequencing conditions within species. Library preparation using poly‐A tail selection took place at each facility for each set of samples prior to sequencing. TruSeq and NEB Next Ultra RNA library prep kits were used at UC Berekely and Novogene, respectively. Cleaning and size selection of reads took place using the AMPure XP system. These data are available on NCBI's Sequence Read Archive (SRA) database under BioProjects PRJNA850087 and PRJNA850454.

After sequencing, read quality was assessed using FastQC (Andrews, 2010). Adapter sequences were trimmed and sequences with an average quality score of less than 25, averaged over four bases, were removed using Trimmomatic (v0.36; Bolger et al., 2014). Subsequently, reads with a length less than 25 base pairs (bp) were also discarded. Trimmed reads were then assembled de novo using Trinity (v2.6.6) software (Grabherr et al., 2011; Haas et al., 2013) on the National Center for Genome Analysis Support's Carbonate computing cluster at Indiana University. In silico read normalization was performed and default minimum contig length and kmer sizes of 200 and 25 bp, respectively, (Grabherr et al., 2011; Henschel et al., 2012) were used. A combined assembly of all stages was produced by pooling trimmed reads for each species to streamline downstream gene identification. Assembly statistics (Haas et al., 2013) and assessments of transcriptome completeness using BUSCO v3.0.2 (Simão et al., 2015) were calculated. BUSCO evaluations were done using a reference set of orthologous groups (*n* = 1013) found across arthropods.

### 2.3 Opsin identification and phylogenetic analysis

Opsin transcripts were identified from the combined assemblies for each species using the Phylogenetically Informed Annotation (PIA) pipeline on the University of California at Santa Barbara Galaxy instance (Speiser et al., 2014). In brief, open reading frames (ORFs) with a length of at least 150 amino acids were predicted from sequenced contigs, and BLAST searches (*e*‐value threshold = 1e‐5) were performed against the Light Interacting Toolkit (LIT), a collection of genes containing published opsin sequences. Finally, significant hits were aligned using MAFFT (Katoh et al., 2002) and placed into a pre‐existing phylogeny to further differentiate between opsin transcripts and closely related proteins.

Transcripts that fell out at a taxonomic position closest to opsin‐like proteins or outgroups, rather than published opsins in the tree generated by PIA, were removed. Remaining sequences were checked to ensure they contained amino acid motifs such as the DRY or QAKKM sections which are involved in G‐protein binding and are conserved within opsins, as well as the chromophore binding lysine at bovine rhodopsin position 296 (Franke et al., 1990; Kӧnig et al., 1989; Park et al., 2008). Sequences with at least 98% similar overlapping nucleotide sequences were combined to create a consensus sequence. Transcriptomes were analyzed with euGenes/EvidentialGene (Gilbert, 2013) which uses gene prediction and assembly software to produce a list of best quality contigs per “locus.” In brief, three datasets were generated from each transcriptome with the tr2aacds4 script to annotate and score gene constructions to reduce redundancy and remove putative isoforms: a “drop set” of redundant or low‐quality coding sequence which likely are artifacts of assembly error, an “okay‐alternative set” which contains potential isoforms, paralogs and mid‐quality coding sequence, and an “okay set” which contains the most complete and unique predicted mRNA transcripts (Gilbert, 2019). Only transcripts identified both by PIA and supported by the “okay set” of euGenes/EvidentialGene were used to generate an opsin phylogeny and for expression comparisons.

Gene expression data were generated by mapping reads from each individual back to the initial combined assembly, prior to filtering with euGenes/EvidentialGene, using RSEM and default parameters (Li & Dewey, 2011). Putative opsin transcripts with an average expression in transcripts per million (TPM) of less than one across developmental stages were treated as the result of contamination and were discarded (Hart et al., 2013; Suvorov et al., 2017). To generate a comprehensive opsin tree, translated putative opsin transcripts and published protein sequences curated by Porter et al. (2011), including placozoan opsin‐like sequences and closely related GPCRs which were used as an outgroup, were compiled into a MAFFT alignment using automatic algorithm selection in Geneious R10 (Katoh et al., 2002; Kearse et al., 2012). Model selection was performed using IQ‐tree (Trifinopoulos et al., 2016) and a protein GAMMA model was selected with a LG4M substitution matrix. Maximum likelihood trees were then inferred using RaxML (Stamatakis, 2014) on the CIPRES platform (Miller et al., 2010) using 1000 bootstrap iterations. The resulting tree was visualized in FigTree (v.1.4.3; Rambaut, 2007) and used to infer the clade each putative opsin belonged to.

A phylogenetic tree generated by the methods described above was created to identify *G. falcatus* sequences homologous to previously published opsins from the closely related species *N. oerstedii* (Bok et al., 2014; Porter et al., 2020). Sequences were determined to be homologous if they formed a monophyletic clade and were at least 90% similar in amino acid sequence. Though opsin localization data were not obtained for *G. falcatus*, the similarity of its opsin sequences to those expressed in *N. oerstedii* adult retinas, as well as the close phylogenetic relationship of these species (Van Der Wal et al., 2017) could provide a clue as to the location of *G. falcatus* opsin expression in the retina.

### 2.4 Identification of spectral tuning sites

Amino acids near the chromophore have been shown to affect the peak wavelength of light a photopigment absorbs at, a phenomenon known as spectral tuning (Chan et al., 1992; Neitz et al., 1991). We compared the amino acid composition of *G. falcatus* opsin sequences at these positions to determine whether the putative photopigments formed by these opsins would likely absorb at different wavelengths. Swiss‐PdbViewer was used to fit a *G. falcatus* opsin transcript (MWS contig 1) to the structure of jumping spider rhodopsin (Varma et al., 2019; PDB ID code 6I9K) which is the most closely related protein structure published at this time, and to identify amino acid residues within 5 Å of the chromophore. Sequences were also aligned with bovine rhodopsin (NCBI accession no. NP_001014890), a commonly used reference, for ease of comparison. Several sites in the sixth and seventh transmembrane helix regions of the opsin were lacking in data, with over 20% of *G. falcatus* opsin transcripts terminating before this point. These sites were not included in the analysis, and the remaining sites were checked for amino acid differences between opsins.

## 3 RESULTS

### 3.1 Opsin diversity

While the *P. thomassini* assembly showed some fragmentation with a BUSCO score of 82.9% the *G. falcatus* assembly appeared relatively complete with a score of 97.1% (see Table S1 for complete assembly statistics). Opsin transcripts from all developmental stages clustered with pancrustacean long (LWS; >500 nm), middle (MWS; ~400–500 nm), and short/ultraviolet (SWS/UVS; <400 nm) wavelength visual opsin clades (Cronin & Porter, 2014; Porter et al., 2007), and previously described stomatopod opsin subgroups (Porter et al., 2009, 2020). No nonvisual opsins were identified from either species.

A total of 27 putative opsin transcripts were found in *P. thomassini* and all were expressed in the adult eye. Embryonic and larval *P. thomassini* stages expressed a subset of the opsin transcripts found in adult eyes with additional opsins being found in later developmental stages and no opsins being lost. Opsin copy numbers progressed from eight in the two earliest embryonic stages to 24 in late‐stage larvae (Figure 1, Table S2 for TPM values).

*Gonodactylaceus falcatus* transcriptomes contained 33 putative opsins. Male and female adults differed slightly in expression with 32 opsin transcripts found in the female eye and 30 in the male eye (Figure 2, Table S2 for TPM values). Two LWS opsin transcripts that were found in the female as well as all other developmental stages were not expressed in the male. Larvae expressed 22 opsin transcripts with one MWS opsin transcript found only in larval and postlarval transcriptomes and all others overlapping with those found in adults. The postlarva was the only developmental stage found to express all 33 opsin transcripts for this species.

In general, a lower copy number of LWS and MWS opsin transcripts were found in embryonic and larval datasets (Figure 1, Figure 2). The number of UVS opsin transcripts was also lower in embryonic *P. thomassini*, with one UVS opsin transcript not expressed in embryos at all, but both UVS opsin transcripts expressed in larval individuals. All three UVS opsin transcripts found in the *G. falcatus* transcriptomes were expressed in both larval and postlarval stages.

### 3.2 Phylogenetic analysis

**FIGURE 3:**
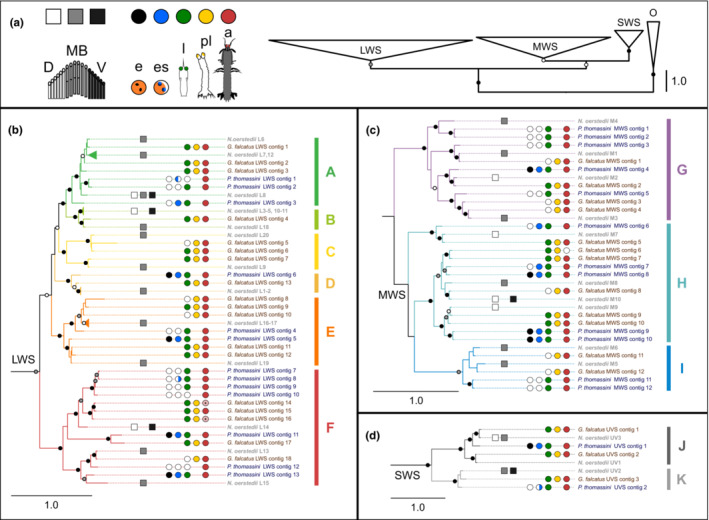
Phylogeny of published *Neogonodactylus oerstedii* opsins (names in gray) with *Pullosquilla thomassini* (names in blue) and *Gonodactylaceus falcatus* (names in brown) transcripts. The phylogeny is broken down into major clades (long [LWS], middle [MWS], and short [SWS] wavelength‐sensitive clades) with a condensed tree showing relationships between clades as well as an outgroup (O) consisting of placozoan opsin‐like sequences. Bars on the right represent opsin subgroups (A–K; Porter et al., 2020). Expression pattern in the retina, if known, is indicated by square boxes for *N. oerstedii* sequences. Presence of each opsin in developmental stages is indicated by colored circles for *P. thomassini* and *G. falcatus*. Bootstrap support is represented with small white circles (70%–79%), gray circles (80%–89%), and black circles (90%–100%). (a) Diagrams of a stomatopod eye with the dorsal (D, white), ventral (V, black), and midband (MB, gray) regions labeled; developmental stages e (embryonic stages 1 and 2, black), es (embryonic stage 3 and propelagic larvae, blue), l (mid and late‐stage larvae, green), pl (postlarva, yellow), and a (adult, red). (b) Phylogeny of long wavelength‐sensitive opsins. *Pullosquilla thomassini* LWS contig 1 was found in embryonic stage 3 but not the propelagic stage while LWS contig 8 was found in the propelagic but not embryonic 3 stages. Both are indicated with half circles. *G. falcatus* LWS contigs 14 and 16 were found in female but not male adults and are indicated by a + sign. (c) Phylogeny of middle wavelength‐sensitive opsins. (d) Phylogeny of short wavelength‐sensitive opsins. *P. thomassini* UVS contig 2 was found in propelagic but not embryonic stages.

Of the 33 putative opsins identified from *G. falcatus*, 20 clustered with previously published *N. oerstedii* opsins including 14 transcripts which were expressed in larvae (Figure 3). Four LWS opsin transcripts found in larval *G. falcatus* clustered with mRNA sequences that have localized expression exclusively in the midband row in adult *N. oerstedii* (NoL6, 9, 18, and 20; Porter et al., 2020). The UV1 and UV2 opsin transcripts found in *G. falcatus* larvae have expression patterns that include midband and peripheral hemisphere R8 cells in adult *N. oerstedii* eyes (Bok et al., 2014).

### 3.3 Analysis of spectral tuning sites

A total of 18 sites within 5 Å of the chromophore with data from at least 80% of *G. falcatus* opsin sequences were identified (Table S3). Thirteen sites showed variation that corresponded with spectral clades or subgroups (Table 1) and included sites with phenylalanine/tyrosine (F/Y) or serine/alanine (S/A) amino acid substitutions which have been found to change peak absorbance of opsins in other species (Chang et al., 1995; Janz & Farrens, 2001; Lin et al., 1998; Salcedo et al., 2009; Takahashi & Ebrey, 2003; Yokoyama et al., 1999; Yokoyama & Yokoyama, 1990). Of the remaining sites, three at bovine rhodopsin positions 121, 181, and 187 were invariable between gene copies and two at bovine rhodopsin positions 114 and 117 had substitutions between amino acids with similar size, polarity, and hydrophobicity (Table S3).

**TABLE 1:** Amino acid positions near the chromophore for which data are present in at least 80% of opsin sequences with variation which corresponds to clade structure.

Position		LWS						MWS			UVS	
MWSc1	BvRh	A (3)	B (1)	C (3)	D (1)	E (5)	F (5)	G (4)	H (6)	I (2)	J (2)	K (1)
110	90	M_1_? _2_	M	M_1_? _2_	M_1_ L_1_? _1_	M	M_4_? _1_	Q	Q	N	K	K
**126**	**113**	**Y**	**Y**	**Y**	**Y**	**Y**	**Y**	**H**_**2**_ **Y**_**1**_ **F**_**1**_	**Y**	**Y**	**Y**_**1**_ **F**_**1**_	**F**
**139**	**118**	**N**	**N**	**N**_**2**_ **K**_**1**_	**N**	**S**	**S**_**4**_ **T**_**1**_	**S**_**2**_ **A**_**2**_	**A**	**G**	**A**	**N**
143	122	C	C	C	C	C	C_4_ T_1_	D	L	V	P	I
**199**	**178**	**Y**	**Y**	**Y**	**Y**	**Y**	**Y**	**Y**_**3**_ **F**_**1**_	**Y**	**Y**	**F**_**1**_ **?**_**1**_	**Y**
**207**	**186**	**A**	**A**	**V**_**2**_ **A**_**1**_	**A**	**A**	**A**_**4**_ **V**_**1**_	**S**	**S**	**S**	**S**_**1**_ **?**_**1**_	**T**
209	188	G	G	G	G	G	G	S	S	S	T_1_? _1_	S
210	189	T	T	T	T	T	T	F	F	F	F_1_? _1_	M
223	204	L	L	L	L	L	L_3_ V_2_	G	G	T	V_1_? _1_	V
**226**	**207**	**Y**	**Y**	**Y**	**Y**	**Y**	**Y**_**4**_ **F**_**1**_	**L**_**3**_ **V**_**1**_	**1**_**5**_ **L**_**1**_	**I**	**L**_**1**_ **?**_**1**_	**T**
**227**	**208**	**T**	**S**	**S**	**A**_**3**_ **S**_**2**_	**A**_**3**_ **S**_**2**_	**T**_**4**_ **V**_**1**_	**F**	**F**	**F**	**F**_**1**_ **?**_**1**_	**F**
230	211	V	V	V	V	V	C	C	D_5_ C_1_	D	V_1_? _1_	A
**231**	**212**	**F**	**F**	**F**	**Y**	**Y**	**Y**_**4**_ **F**_**1**_	**F**_**2**_ **Y**_**2**_	**Y**_**5**_ **?**_**1**_	**Y**	**Y**_**1**_ **?**_**1**_	**Y**

*Note*: Positions identified by *Gonodactylaceus falcatus* MWS contig 1 (MWSc1; NCBI accession no. OP066265) and Bovine rhodopsin (BvRh: NCBI accession no. NP_001014890.1). Opsins are divided by spectral clades and subgroups with the number of sequences in the subgroup in parenthesis. Within each subgroup, the amino acid identity at each position is given. For subgroups where more than one amino acid is found at the given position the number of sequences with a particular residue is indicated by subscript. Positions where F/Y or S/A substitutions occur are bolded. Question marks indicate unknown amino acids at a specific position. See Table S2 for additional information.

## 4 DISCUSSION

Our results show that while larval stomatopods lack the extensive opsin repertoire of their adult counterparts, they do possess the molecular components to detect light across the visible spectrum. While previous MSP work, which quantifies absorbance of photopigments, suggested the existence of a single middle wavelength absorbing visual pigment in larval stomatopod retinas (Cronin et al., 1995; Feller & Cronin, 2016; Jutte et al., 1998), this study shows that stomatopod larvae are capable of expressing multiple opsin transcripts, implying the existence of multiple photopigments and opening the possibility of multiple photoreceptor types. Based on transcriptomic expression we also suggest that the opsin repertoire likely changes throughout stomatopod development and provide evidence that opsins are expressed in developing embryos.

There are two points in the development of *P. thomassini* when the total number of opsin transcripts expressed shifts between stages (Figure 1). The first two embryonic stages have the same reduced opsin repertoire, but the number of MWS and LWS opsin transcripts expressed increases at embryonic stage 3, coinciding with the development of eyeshine and twitching movement. The third embryonic stage differs from the propelagic larval stage only in the expression of a different LWS opsin and the addition of a UVS opsin transcript. Propelagic *P. thomassini* larvae are negatively phototaxic immediately after hatching (Dingle, 1969), and our findings provide some evidence that the ability to detect light could begin prior to hatching. This would make sense as the ability for propelagic larvae to remain in their parental burrow until they reach a developmental stage at which they are capable of feeding themselves is paramount to their survival. The expression of opsins alone does not confirm the existence of a functional phototransduction pathway at these early life stages (Pérez‐Moreno et al., 2018), but the increase in the number of opsin transcripts being expressed could point to a physiological and developmental shift in the sensory system.

There is another major increase in opsin transcript numbers when moving from the benthic propelagic to the pelagic last larval stage in which nearly as many opsin transcripts per spectral class are expressed as in the adult stage (except for the LWS). This transition may appear less abrupt with the addition of intermediate larval stages which were not sampled here, but the shift from relying on yolk and maternal care to hunting and avoiding predation is a significant one: both ecologically and in terms of the behavioral cues provided by light detection. Based on these data, it appears that increases in opsin expression are correlated with increasing diversity of habitats and behaviors experienced by developing stomatopods. The ability to distinguish a wider array of wavelengths may be advantageous as larvae move from performing a simple task, staying within the parental burrow, to hunting and avoiding predators in the pelagic realm.

It is important to note that while opsin mRNA does imply transcription of opsin genes, the translation of these transcripts into a functional protein has yet to be verified. Opsin transcript expression data (Table S2) was consistent with use in light detection (Chauhan et al., 2014; Lowe et al., 2018); however, antibody staining is still needed to confirm the presence of functional photopigments in embryonic and larval stomatopods, as well as the additional components needed for phototransduction. The absence of specific opsin transcripts in a particular developmental stage is not possible to prove with our current datasets, though the inclusion of RNA from multiple individuals in embryonic and larval transcriptomes does provide a measure of certainty. While sampling more individuals would be advantageous to provide statistical evidence of the trends we see here, previous work has demonstrated that opsin transcript expression in larval stomatopods is relatively consistent across individuals (McDonald et al., 2022).

The large increase in the number of opsins expressed between the propelagic and adult developmental stages in *P. thomassini* prompted a more detailed investigation of the transition from larval to adult photosensory system. Because the *P. thomassini* dataset lacked mid‐stage larval and postlarval representatives and was sequenced at an earlier time using less precise technology, filling in data for the missing stages and production of less fragmented transcriptomes were the focus of the *G. falcatus* dataset. The identification of opsin transcripts in larval stomatopods using our *P. thomassini* dataset was further supported by our more complete *G. falcatus* transcriptomes. The close phylogenetic relationship between *G. falcatus* and the well characterized *N. oerstedii* also allowed us to speculate with greater certainty about a potential use in vision for the putative opsins described here. The most unexpected finding from this comparison was the expression of orthologs of adult *N. oerstedii* opsins L6, L9, L18, and L20, in larval *G. falcatus*. These LWS visual opsins have expression patterns confined to the midband of adult *N. oerstedii* eyes (Porter et al., 2020). If we infer similar expression patterns in adult *G. falcatus*, finding these transcripts in life stages that lack a midband is perplexing and necessitates further study, especially in the case of L9 and L18 which had very high expression (Table S2).

While larval stomatopod retinas were at one point thought to be spatially homogenous (Cronin et al., 1995; Jutte et al., 1998), data from at least one family, the Nannosquillidae, provide evidence of regional localization of intrarhabdomal structural reflectors that may tune photoreceptors in the lateral and ventral portions of the eye (Feller et al., 2019). If expressed in the eye, the existence of multiple photopigments could allow for multiple photoreceptor types and potentially differentiation of eye regions. The opsin transcripts found in *P. thomassini*, a nannosquillid, were less similar at the amino acid level to those from *N. oerstedii*, though some did form well supported monophyletic clades with opsins known to be expressed in the midband of the eye (Figure 3). It is not possible to make inferences regarding the location of opsin expression in this species, as it is phylogenetically and ecologically distant from *N. oerstedii*, but localization patterns that differ between opsins could be possible here as well. Further studies on lysiosquilloids are needed to better understand if expression patterns and opsin sequence identity are comparable across superfamilies.

Because embryonic, larval, and postlarval RNA was generated from whole bodies or heads, rather than exclusively from eyes, the use of identified putative opsins in vision cannot be confirmed. However, given the number of opsins expressed in the larvae of *P. thomassini* and *G. falcatus*, the use of only one photopigment in vision seems unlikely. Further mRNA and protein localization studies are needed to determine whether opsin expression is confined to embryonic and larval eyes, or if the extraocular expression of so‐called “visual” opsins may also be occurring. Because RNA from adults was isolated from eye tissue, and sequences were phylogenetically similar to opsins expressed in the retina of *N. oerstedii* (Porter et al., 2020), a stronger case can be made for the use of these putative opsins in vision. Further work is still necessary to ascertain that these transcripts are the precursors to functional proteins and confirm localization in the retina.

It is unclear whether the large number of putative opsins found here are the result of recent duplication, due to a lack of reference genome to base this on. No stomatopod genomes have been sequenced yet, but data from other pancrustaceans point towards a large genome (Colbourne et al., 2011; Jeffery et al., 2017; Poynton et al., 2018; Zhang et al., 2019), and a large number of opsin sequences with documented expression from this order suggest rampant gene duplication and high levels of sequence similarity between opsins (Cronin et al., 2010; Porter et al., 2009, 2020). Both of these factors present issues when assembling transcriptomes and though we have been conservative in our estimation we cannot completely discount the possibility that some of the described opsin transcripts may be isoforms of a single opsin. Even if opsin transcripts which form monophyletic clades within a species (e.g., *P. thomassini* LWS contigs 1 and 2) are regarded as potential isoforms, all developmental stages from both species sampled here expressed putative opsins from multiple previously described and well supported stomatopod opsin clades (A–H, Figure 3; Cronin et al., 2010, Porter et al., 2020).

Variation in the amino acid composition of opsin proteins can provide information about the peak absorbance wavelength of the photopigment it would form. Peak light absorbance measurements taken using MSP from adult *G. falcatus* retinas at the corresponding midband (MB) rows where opsins L6, L9, L18, and L20 would be expressed (MB 5–6; MB 1–4; MB 5–6; MB2 proximal tier and MB3) range from 400 to 551 nm (Cronin et al., 1995). However, multiple opsins have been found to be co‐expressed in the midband photoreceptors of *N. oerstedii* (Porter et al., 2020), making the peak absorption of individual photopigments impossible to determine using retinal absorbance alone. The variation found in amino acid residues near the chromophore, particularly the existence of S/A and F/Y substitutions between spectral clades (LWS, MWS, and UVS), provides strong evidence that the absorption of the photopigments formed with these opsins would differ if they were translated into functional proteins (Chang et al., 1995; Janz & Farrens, 2001; Lin et al., 1998; Salcedo et al., 2009; Takahashi & Ebrey, 2003; Yokoyama et al., 1999; Yokoyama & Yokoyama, 1990). In our data, variation is also found in opsin subgroups, particularly within MWS subgroups H and G. Larval *G. falcatus* express opsin transcripts from every subgroup except MWS subgroup I, meaning that the amino acid variation observed between spectral clades is present in the larval data, as well as in the postlarval and adult stages (Figure 3).

The ability to distinguish between multiple wavelengths of light may provide behavioral advantages to stomatopod larvae. Instances of arthropods relying on specific wavelengths of light as cues for certain behaviors exist in the marine realm and beyond (Phillips & Sayeed, 1993; Scherer & Kolb, 1987; Stukenberg et al., 2020). For example, the water flea *Daphnia magna* has been shown to use UV wavelengths as a cue for vertical migration (Storz & Paul, 1998) while the amphipod *Talorchestia longicornis* relies on blue light cues for orientation (Forward et al., 2009). It is therefore conceivable that larval stomatopods may utilize different light cues for behaviors such as prey detection, orientation in the water column, and settlement. Visual detection of and differentiation between wavelengths provides advantages such as the ability to effectively discriminate prey from the background in marine environments (Marshall et al., 2015).

Cataloging opsin expression across developmental stages is an important step towards understanding how light detection may shift at different points in the stomatopod life cycle. While this study does not focus specifically on visual capabilities, the expression of numerous opsin transcripts in larval stomatopods does not suggest that only one photopigment is present in early stomatopod life stages (Cronin et al., 1995; Feller & Cronin, 2016; Jutte et al., 1998). These data instead suggest the presence of multiple photopigments and open up the possibility of multiple visual and/or nonvisual photoreceptor types in larval stomatopods across species. The existence of multiple functional photopigments in the eyes of one species of larval stomatopod has already been confirmed by electrophysiological experiments in which both a UV and blue spectral peak were detected in the eyes of *N. oerstedii* larvae (McDonald et al., 2022). While more work is needed to determine whether the putative opsins found here do indeed form functional photopigments, it seems likely that future studies of larval stomatopod photoreception will uncover additional light detection abilities spanning the visual spectrum.

## AUTHOR CONTRIBUTIONS

**Sitara Palecanda:** Conceptualization (equal); data curation (lead); formal analysis (equal); investigation (lead); methodology (lead); project administration (equal). **Mireille Steck:** Data curation (supporting); formal analysis (equal); investigation (supporting); methodology (supporting); project administration (supporting). **Megan L. Porter:** Conceptualization (equal); funding acquisition (lead); investigation (supporting); methodology (supporting); project administration (equal).

## CONFLICT OF INTEREST STATEMENT

The authors declare no competing interests.

## FUNDING INFORMATION

Transcriptome sequencing was funded by the University of Hawaiʻi at Mānoa. Computing resources were supported by National Science Foundation grants DBI‐1458641 and ABI‐1062432 to Indiana University.

## Supporting information


Table S1

Table S2

Table S3

## Data Availability

mRNA sequences have been submitted to NCBI's GenBank and accessioned OP066247‐OP066306. Full RNAseq datasets have been submitted to NCBI's SRA database and given BioProject IDs PRJNA850087 and PRJNA850454.
