# Supplementary material for: Increasing complexity of opsin expression across stomatopod development
Source: Ecol Evol. 2023 May 26;13(5):e10121. doi: 10.1002/ece3.10121 (PMC10220389; doi:10.1002/ece3.10121)
Supplement: Supplementary file 1 — Table S1 [file ECE3-13-e10121-s001.docx]

**Supplementary table S1.** De novo transcriptome assembly statistics from combined *P. thomassini* (Pt) and *G. falcatus* (Gf) assemblies.

| **Assembly** | **Number of transcripts** | **Mean transcript length (bp)** | **N50 (bp)** | **Complete single copy BUSCOs** | **Complete duplicated BUSCOs** | **Fragmented BUSCOs** | **Missing BUSCOs** |
| --- | --- | --- | --- | --- | --- | --- | --- |
| Pt combined | 470,769 | 489 | 581 | 15% | 67.9% | 11.5% | 5.6% |
| Gf combined | 767,962 | 558 | 773 | 32.6% | 64.5% | 2.2% | 0.7% |
